# Quercetin as a Potential Therapeutic Agent for Malignant Melanoma—A Review of Current Evidence and Future Directions

**DOI:** 10.3390/medicina61040656

**Published:** 2025-04-02

**Authors:** Teodora Hoinoiu, Victor Dumitrascu, Daniel Pit, David-Alexandru Schipor, Madalina Jabri-Tabrizi, Bogdan Hoinoiu, David Emanuel Petreuș, Corina Seiman

**Affiliations:** 1 Department of Clinical Practical Skills, “Victor Babes” University of Medicine and Pharmacy, Eftimie Murgu Sq. Nr. 2, 300041 Timisoara, Romania; tstoichitoiu@umft.ro (T.H.); madalina.plic@umft.ro (M.J.-T.); 2 Center for Advanced Research in Cardiovascular Pathology and Hemostaseology, “Victor Babes” University of Medicine and Pharmacy, 300041 Timisoara, Romania; 3 Department of Pharmacology, “Victor Babes” University of Medicine and Pharmacy, 300041 Timisoara, Romania; dumitrascu.victor@umft.ro; 4 Doctoral School, “Victor Babes” University of Medicine and Pharmacy Timisoara, E. Murgu Square, No. 2, 300041 Timisoara, Romania; corina.duda@e-uvt.ro; 5 Faculty of Chemistry, Biology, Geography, West University of Timisoara, Pestalozzi 16, 300115 Timisoara, Romania; schiporalexandrudavid@yahoo.com; 6 Department of Oral Rehabilitation and Dental Emergencies, Faculty of Dentistry, “Victor Babes” University of Medicine and Pharmacy, P-ta Eftimie Murgu 2, 300041 Timisoara, Romania; hoinoiu@umft.ro; 7 Interdisciplinary Research Center for Dental Medical Research, Lasers and Innovative Technologies, 300070 Timisoara, Romania; 8 Department of Chemistry, Faculty of Chemistry, Biology, Geography, West University of Timisoara, Pestalozzi 16, 300115 Timisoara, Romania

**Keywords:** skin cancer, quercetin, melanoma, wound healing

## Abstract

Neoplastic disorders, particularly malignant carcinomas, are complex systemic diseases characterized by unregulated cellular proliferation, the invasion of adjacent tissues, and potential metastasis to distant bodily sites. Among the diverse spectrum of cancer subtypes, malignant melanoma is a highly aggressive form of cutaneous cancer originating in melanocytes, the pigment-producing cells resident in the skin. This malignancy is distinguished by its rapid and uncontrolled growth, as well as its propensity for metastasis to vital organs, thereby posing significant challenges to therapeutic intervention and prognostication. Early detection of melanoma is crucial for optimizing patient outcomes, as diagnosis at an advanced stage often yields a poor prognosis and limited treatment options. Diagnostic modalities for melanoma encompass comprehensive clinical evaluations by dermatologists; radiological imaging techniques such as ultrasonography, magnetic resonance imaging (MRI), computed tomography (CT) scans; and excisional biopsies for accurate histopathological assessment. Malignant melanoma is typically treated with surgery to remove the tumor, followed by immunotherapy to enhance the immune response, targeted therapy for tumors with specific genetic mutations, chemotherapy for advanced stages, radiation therapy to manage metastasis, and other adjunct therapies. This review presents the properties and possible adjunct therapeutic effects against malignant melanoma of quercetin found in the literature and explores, based on the observed physicochemical properties and biological activity, its potential development as a topical formulation for cutaneous application. Quercetin is a naturally occurring flavonoid compound abundant in various plant-based food sources, including apples, onions, berries, and citrus fruits, and has exhibited promising antiproliferative, antioxidant, and anticancer properties. Its distinctive biochemical structure enables quercetin to effectively neutralize reactive oxygen species and modulate key carcinogenic pathways, thereby rendering it a potential candidate for therapeutic intervention in managing malignant tumors, including melanoma.

## 1. Introduction

A neoplasm is a complex systemic disorder characterized by the unregulated proliferation of malignant cells, intricate reciprocal interactions with adjacent cells, the invasion of healthy tissues, and dissemination throughout the body via metastatic processes under systemic regulatory mechanisms. In its advanced stages, cancer ultimately leads to human mortality through metastasis, which occurs via the vascular and lymphatic systems. The human body relies on a delicate interplay among diverse tissues and organs, with each organ executing its specialized function through the agency of tissue-specific cellular networks [1].

Cancer can be broadly categorized into four primary types: carcinoma, sarcoma, melanoma, and lymphoma [2]. Neoplasms constitute the second leading cause of mortality in developed nations. The high mortality rate can be attributed to the propensity for cancer to be diagnosed at an advanced stage, thereby diminishing the prospects of survival. Timely detection is essential for enhanced survival outcomes, and delayed diagnosis can lead to severe consequences [3,4]. Due to the absence of early symptoms and patients’ reluctance to seek medical evaluation, most cases are diagnosed late. As a result, survival rates are contingent upon the initial stage at which the disease is detected [5,6].

Cutaneous melanoma is a malignant tumor derived from melanocytes and is one of the most aggressive and lethal forms of skin cancer. Although it accounts for only about 4% of all cutaneous neoplasms, it is responsible for 80–90% of skin cancer deaths, highlighting its high metastatic potential and the need for effective therapeutic strategies [3,4,5,6]. Melanoma is typically treated with surgery to remove the tumor, followed by immunotherapy to enhance the immune response, targeted therapy for tumors with specific genetic mutations, chemotherapy for advanced stages, radiation therapy to manage metastasis, and other adjunct therapies [6,7].

Among potential adjunct therapies, the substance quercetin, a naturally occurring flavonoid found in numerous plant-based foods, including apples, onions, berries, and citrus fruits, has garnered attention for its promising biological activities, such as its antiproliferative, antioxidant, and anticancer properties [8]. Recent studies have highlighted its ability to inhibit key signaling pathways involved in cancer progression, including those related to inflammation, angiogenesis, and metastasis, which are critical in melanoma pathogenesis [8]. Quercetin’s ability to modulate oxidative stress and its diverse anticancer properties make it a highly relevant compound for further investigation in malignant melanoma. Given its widespread availability in dietary sources and relatively low toxicity, quercetin may not only complement current melanoma therapies but also serve as a preventive measure, potentially offering new avenues for the management of this aggressive skin cancer [8].

This review discusses general information about malignant melanoma and the mechanisms by which quercetin may act synergistically with standard treatments for this condition. Additionally, we address its bioavailability, stability, and the feasibility of developing a topical cream or other cutaneous formulation. These convenient and non-invasive formulations provide patients with reassurance and comfort. We explore the pharmacokinetic properties that would support its topical application.

## 2. Methods

A systematic review of the literature published in the last 10 years (2015–2025) was performed to investigate the relationship between quercetin and melanoma. Searches were conducted in PubMed and SCOPUS databases using the following strategy: (“quercetin” AND “melanoma”) and (“quercetin” AND “melanoma” AND “skin cancer”).

After querying the SCOPUS database, 471 articles were identified for the first combination of terms and 101 articles for the second (Table 1). PubMed database searches returned 131 articles for the first strategy and 51 for the second (Table 1). To avoid redundancies, references in the two databases were compared, and duplicate articles were removed. The study included only the articles remaining from this selection process, thus ensuring a rigorous and relevant analysis of the available literature.

## 3. Results

### 3.1. Assessing and Diagnosing Melanoma Types

A comprehensive range of paraclinical investigations is recommended to thoroughly investigate the type of melanoma in a patient presenting suspicious symptoms. Paraclinical investigations of malignant cutaneous melanoma include a complete set of general blood tests, such as a full blood count and differential count; general urine tests, including urinalysis and urine sediment examination; blood biochemistry tests, comprising total protein, total bilirubin and its fractions, urea, creatinine, aminotransferases (including aspartate aminotransferase and alanine aminotransferase), and alkaline phosphatase; a coagulation panel, which encompasses prothrombin time, activated partial thromboplastin time, and fibrinogen level; an electrolyte panel, which includes sodium, potassium, chloride, and calcium levels; an electrocardiogram (ECG) to assess cardiac function; determination of blood type and Rh factor to facilitate potential transfusions; and screening tests for syphilis, such as the rapid plasma reagin (RPR) test, and human immunodeficiency virus (HIV) infection to identify potential co-infections [9].

Investigations to comprehensively determine the extent of malignant cutaneous melanoma, also known as clinical staging, involve a thorough and objective physical examination by a medical professional [10]. Radiological examinations, including imaging tests such as computed tomography (CT) scans, magnetic resonance imaging (MRI) scans, and positron emission tomography (PET) scans, are also conducted to assess the spread of the disease. A chest X-ray is typically performed to evaluate the lungs and surrounding tissues for any signs of metastasis or abnormal growth. Mediastinal tomography, a specialized imaging test, examines the mediastinum, the central part of the chest cavity, for any abnormalities or cancer spread. An ultrasound examination of abdominal cavity organs, such as the liver, spleen, and kidneys, is also conducted to check for any signs of metastasis or cancer spread to these organs. X-rays of bones are usually performed in the presence of bone pain or other symptoms suggesting bone metastasis to assess the extent of cancer spread to the skeletal system [9,10].

To establish a definitive and accurate diagnosis of malignant cutaneous melanoma, performing a thorough cytological smear directly from the ulcerated tumor is essential, as well as carefully collecting and examining the cells for abnormal characteristics. An excisional biopsy of the cancer, which involves surgically removing the entire tumor along with a margin of surrounding healthy tissue, is also a crucial diagnostic approach that provides a comprehensive understanding of the tumor’s histopathology [11]. Alternatively, a radiographic examination of the affected area, such as an X-ray or CT scan, may be warranted in some instances to assess the extent of the tumor’s growth and potential spread to other parts of the body [9,10,11].

This involves comprehensive consultation in specialized dermatological centers, where a thorough and meticulous skin examination and detailed nevus (mole) assessment are performed. Nevi are carefully examined using the advanced mapping method, which involves precise microscopic analysis and state-of-the-art digital monitoring of the moles [11]. A comprehensive examination of patients is conducted, encompassing a meticulous evaluation of all existing moles from head to toe. High-resolution photographs of these moles are captured utilizing a state-of-the-art, high-performance camera, and a detailed topological map of their locations is subsequently created [12,13]. Employing advanced video dermoscopy, moles are subjected to microscopic analysis with enhanced precision, facilitating a more accurate assessment. This cutting-edge technology enables surface and shallow-depth microscopy, permitting the careful observation and evaluation of potential malignancy in suspicious moles or cellular clusters exhibiting abnormal behavior [11,12,13,14].

**Figure 1:**
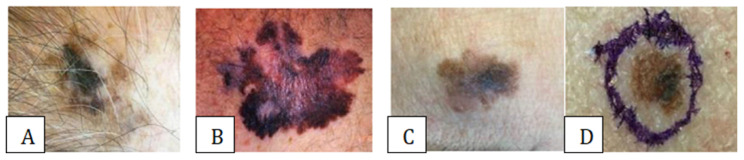
Visible characteristics for melanoma identification [14]. (**A**)**—Asymmetry:** If a line is drawn through the middle of the lesion and the two sides are not identical (asymmetrical), this could be a warning sign for melanoma [14]. (**B**)**—Border:** The edges of melanoma tend to be uneven. Irregular, unclear, or notched edges are suspected of melanoma [14]. (**C**)**—Color:** A benign mole is a single color. Multiple colors in a skin lesion may indicate melanoma [14]. (**D**)**—Diameter:** Moles larger than 6 mm in diameter may suggest melanoma [14].

The suspicious moles are assessed following the widely recognized “ABCD” criteria (Figure 1), a standardized framework established by esteemed experts Nachbar and Stolz. This comprehensive evaluation involves examining the mole for notable asymmetry of shape, where one half of the mole does not exhibit symmetry with the other half. Another crucial diagnostic feature is that border irregularity is characterized by indistinct, irregular, and uneven edges that fail to provide a clear demarcation between the mole and the surrounding skin. Color variegation, where the mole exhibits multiple shades or hues in different areas, is also a key consideration in the analysis. The mole’s diameter, or size, is the final factor considered when thoroughly examining suspicious moles [14].

### 3.2. Quercetin

Quercetin is a naturally occurring, bioactive compound commonly found in various plants. It exhibits potent anti-inflammatory and antioxidant properties that have garnered it significant attention for its potential health benefits. It may help reduce chronic inflammation and accelerate the healing process of wounds, burns, and scars by promoting tissue repair and regeneration, thereby improving overall health outcomes [8,15,16,17,18,19,20,21,22].

**Figure 2:**
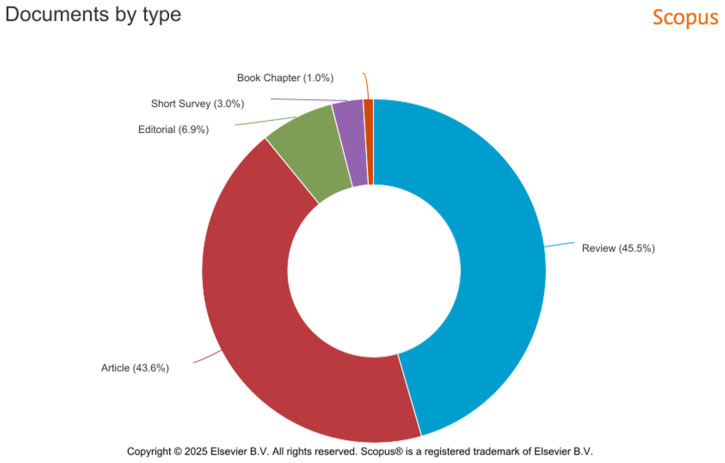
Distribution of document types identified in the Scopus database following the literature review.

The graph in Figure 2 shows the distribution of document types related to quercetin and melanoma in the Scopus database. The most frequent publication types are reviews, representing 45.5% of the total, followed by articles, with 43.6%. This distribution suggests that the field benefits from extensive critical analysis, with many review articles evaluating the existing literature. The high proportion of reviews indicates increasing interest in consolidating and interpreting the data on the effects of quercetin in treating melanoma.

Quercetin is a secondary polyphenolic metabolite belonging to the flavonoid class, distinguished by its benzo-(γ)-pyrone skeletal configuration with a C6-C3-C6 carbon framework, comprising two benzene rings, A and B, connected by a pyrone ring (C) with three carbon atoms. The compound is called a pentahydroxyflavonol due to five hydroxyl groups on its flavonol skeleton at carbon atoms 3, 3′, 4′, 5, and 7. The extensive range of biochemical and pharmacological activities exhibited by quercetin and its metabolites can be attributed to the diverse substitution patterns observed in the functional groups present in the flavonol molecule. Quercetin is a yellow crystalline solid with a bitter taste that is insoluble in water and slightly soluble in alcohol, glacial acetic acid, and aqueous alkaline solutions [8,15]. It is a member of a class of naturally occurring compounds known as flavonoids, which feature a common flavone nucleus comprising two benzene rings connected by a heterocyclic pyrone ring. As animals cannot synthesize the flavone nucleus, flavonoids are found exclusively in plants. Quercetin is a key member of the polyphenol family and is predominantly found in various vegetables and fruits, such as capers, lovage, dill, coriander, onions, multiple berries (e.g., chokeberries, blueberries), and apples [8,15].

## 4. Discussions

Flavonoids are a prevalent natural occurrence, predominantly present in benzo-γ-pyrone derivatives. These compounds are predominantly found in various plants, vegetables, and floral species. Flavonoids exhibit a wide range of structural diversity and serve pivotal functions within the body’s defense system. The beneficial effects of flavonoid-rich foods have been demonstrated in several studies. Flavonoids are a diverse group of compounds found in nature, with over 4000 different types, which can be further subdivided into various subcategories, including flavones, isoflavones, flavanones, and chalcones. Flavonoids have been demonstrated to possess important biological activities, including anti-inflammatory, antioxidant, hepatoprotective, and antimicrobial properties [8,15,16,17].

Quercetin exhibits a unique capacity to neutralize highly reactive species, including hydrogen peroxide, superoxide anion, and hydroxyl radicals, collectively called reactive oxygen species (ROS). These ROS can potentially cause oxidative damage to cellular components, including proteins, lipids, and deoxyribonucleic acid (DNA). Various oxygen radicals play a significant role in the pathophysiological and degenerative processes associated with aging [8].

In recent years, the treatment of malignant melanoma has evolved significantly, and the introduction of immune checkpoint inhibitors and targeted therapies marked a significant paradigm shift in disease management. While surgical excision remains the primary treatment method, systemic treatments such as PD-1 and CTLA-4 inhibitors, BRAF/MEK-targeted agents, and, in some cases, chemotherapy or radiation therapy play a crucial role in managing malignant melanoma. Ongoing research into combination therapies, oncolytic viruses, and adjunctive natural compounds continues to expand the therapeutic landscape [23]. Quercetin is one of the adjunctive natural compounds that is currently in the limelight, and it has also been extensively researched for its preventive properties.

Using local therapeutic agents in malignant melanoma offers several significant advantages compared to systemic treatments. Local therapy allows for the direct application of the therapeutic agent to the affected site, ensuring high drug concentrations at the tumor site while minimizing systemic exposure, which reduces the risk of adverse effects. Topical and localized formulations can penetrate the melanoma lesion directly, allowing for higher bioavailability of the active compound within the tumor. This is particularly advantageous in early-stage or cutaneous melanoma, where local treatments can efficiently reach cancerous cells while preserving healthy surrounding tissue. Compared to expensive biologics and systemic chemotherapies, local therapies, such as topical formulations, may offer a cost-effective alternative with easier accessibility in low-resource settings. This is particularly relevant in regions where advanced systemic treatments may be unaffordable or unavailable. Quercetin, a naturally available compound, would be easier to obtain and could be incorporated into a healing cream as an adjunct therapy.

Quercetin is recognized as a crucial and naturally occurring cancer-preventing agent among polyphenols. The significance of dietary quercetin lies in its antioxidant potential and anti-inflammatory effects [8]. The preventive and therapeutic effects of quercetin have been extensively demonstrated through experimental discoveries both in vitro and in vivo. When administered in pharmacologically safe doses, Quercetin inhibits the phosphatidylinositol 3-kinase (PI3K)-Akt/PKB (protein kinase B) pathway in cancer cells. Various in vitro studies have consistently shown that quercetin is pivotal in cancer prevention and tumor suppression in diverse cell lines [8]. The anticancer effects of quercetin in vitro have been observed at concentrations ranging from 3 to 50 µM. The cancer-preventing properties of quercetin in vivo have been confirmed in colon cancer models, and its anticancer effects have also been demonstrated in melanoma. The inhibitory effects of quercetin on tumor growth have been evaluated when administered as a dietary supplement in experimental models [8].

Quercetin’s physico-chemical properties—including its ability to neutralize reactive oxygen species (ROS) and modulate key transcription factors such as NF-kB and AP-1—suggest its potential as a topical formulation for the treatment of melanoma. Due to its natural antioxidant properties, quercetin can reduce oxidative stress within the skin, a key contributor to melanoma progression, and may aid in protecting surrounding healthy tissue from the harmful effects of UV radiation, a significant risk factor for melanoma development [24]. Furthermore, quercetin’s ability to influence molecular pathways involved in cell cycle regulation, apoptosis, and immune modulation presents it as a promising candidate for enhancing the effectiveness of existing therapies in melanoma management [24].

Nguyen et al. [25] have investigated the anticancer effects of quercetin on triple-negative breast cancer (TNBC) cells. Quercetin was observed to reduce TNBC cell viability in a time- and dose-dependent manner. The treatment led to increased apoptosis and induced cell cycle arrest. Mechanistically, quercetin enhanced the expression of FasL mRNA and activated signaling pathways involving p51, p21, and GADD45. Additionally, it promoted the protein expression, transcriptional activity, and nuclear translocation of Foxo3a, a transcription factor. The knockdown of Foxo3a significantly diminished quercetin’s effects on apoptosis and cell cycle arrest, highlighting its crucial role in mediating these processes [25].

Hashemzaei et al. [26] proved that quercetin triggers apoptosis in various cancer cell lines, colon carcinoma CT-26 cells, prostate adenocarcinoma LNCaP cells, human prostate PC3 cells, pheochromocytoma PC12 cells, estrogen-receptor-positive breast cancer MCF-7 cells, acute lymphoblastic leukemia MOLT-4 T-cells, human myeloma U266B1 cells, human lymphoid Raji cells, and ovarian cancer CHO cells. Additionally, treatment with quercetin led to a significant increase in survival rates and a substantial reduction in tumor volume in tumor-bearing animals. However, they observed no significant difference in survival rate in the group treated with 50 mg/kg of quercetin or the control group when compared with that of the groups treated with 100 mg/kg and 200 mg/kg. Still, the measured apoptosis for those treated with 50 mg/kg was more significant than the control [26].

In the study conducted by Ren et al. [27], the effect of different doses of quercetin on the growth of ovarian cancer SKOV-3 cells was investigated. The results showed that quercetin inhibited ovarian cancer cell growth. The study also showed that the quercetin-induced cell proliferation inhibition rate significantly increased with the dose and time administered [27].

Seo et al. [20] studied the effect of quercetin on melanoma cells and concluded that it induces mitochondrial apoptosis and downregulates ganglioside GD3 expression through the inhibition of the FAK/paxillin/Akt signaling pathway, which may limit cancer cell survival and metastatic potential [20].

These studies show the possible application of quercetin as an adjunct therapeutic agent for malignant melanoma.

**Figure 3:**
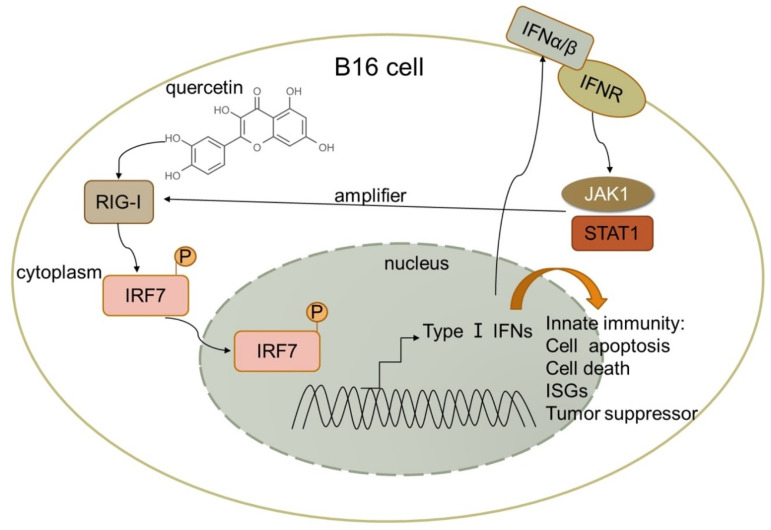
Quercetin mechanism of action on malignant melanoma cell B16 [16].

The table above (Table 2) shows the multiple mechanisms of action for quercetin depending on the type of cancer. For example, by testing the effects of quercetin on two malignant melanoma cells, B16 and A375, one study proved that quercetin upregulated IFN-α and IFN-β expression by activating RIG-I promoter in B16 cells. RIG-I likely amplifies antitumor effects by activating signal transduction and activator of transcription 1 (STAT1) in the IFN-JAK-STAT pathway in an autocrine and paracrine manner (Figure 3). This results in quercetin-induced cell apoptosis and tumor suppression [16].

Besides the main active ingredient, quercetin, a healing cream has to contain specific excipients that work synergistically to enhance the therapeutic effect of the main active ingredient. It also has to be readily available and low-cost. The proposed healing cream contains glycerin, a humectant and skin conditioning agent; petroleum jelly, an occlusive emollient; ethylenediaminetetraacetic acid (EDTA), a chelating agent; and a stabilizer.

Glycerin is a highly effective humectant, meaning it can attract and retain water in the skin, helping maintain optimal moisture levels and supporting the skin’s natural hydration process [31]. This exceptionally beneficial ingredient helps keep the skin incredibly soft and smooth, significantly restoring and rejuvenating the skin’s vital protective barrier. It is also helpful in preventing possible adverse reactions in skin caused by quercetin. Regarding melanoma, the glycerin used in this healing cream plays a crucial role in maintaining the hydration of skin affected by surgical or radiotherapy treatments associated with melanoma, helping to alleviate dryness and discomfort. This can help speed up the healing process, improve the appearance of scars, and enhance overall skin health and well-being [31]. Glycerin’s hydrating effects also enhance the skin’s permeability, allowing easier absorption of the active ingredient quercetin.

Petroleum jelly is a highly effective emollient that acts as a protective barrier on the skin’s surface, safeguarding against dryness and discomfort. It can help retain skin moisture and prevent excessive water loss while providing adequate lubrication, reducing friction and irritation, and promoting overall skin health and well-being [32]. In the case of melanoma, petroleum jelly is used to keep the skin hydrated and supple, especially in areas affected by surgery or treatments associated with melanoma, such as chemotherapy or radiation therapy. It can also help protect the skin from environmental factors such as harmful solar radiation, including UVA and UVB rays, which can exacerbate skin damage and increase the risk of skin cancer [32]. Petroleum jelly is a highly effective ingredient for combating dryness. It is remarkably waxy with a notably denser texture, making it somewhat challenging to use and uncomfortable for general application, especially on large body areas. It has various beneficial effects, such as providing protection, acting as an anti-wrinkle agent, serving as an anti-scratch barrier, and offering moisturizing properties. It can also help prevent skin irritation, remove unsightly scars, and thoroughly nourish the skin [32].

EDTA, also known as ethylenediaminetetraacetic acid, is a compound with exceptional chelating properties, enabling it to effectively bind and inactivate metal ions commonly present in various cosmetic products [33]. This ingredient can significantly help prevent product deterioration through oxidation, thereby contributing to the stability and prolonged effectiveness of active compounds that are often used in skincare formulations, such as quercetin. However, there is no substantial scientific evidence to suggest that EDTA has specific benefits or therapeutic effects in the treatment of melanoma, a type of skin cancer [33]. EDTA undergoes renal elimination in the body and is employed clinically to assess glomerular filtration rates in pediatric populations. Due to its iron-chelating properties, EDTA is an additive in topical agents designed to protect against radiation-induced skin damage, such as antiphotoaging formulations. Furthermore, EDTA is a potent inhibitor of matrix metalloproteinases, rendering it a crucial component in the design of pharmaceutical agents [33]. EDTA can also significantly inhibit the therapeutic effect of bleomycin and has been consistently shown to prevent the nucleolytic activity of bleomycin, a crucial process for bleomycin’s ability to induce apoptosis in rapidly dividing cancer cells. EDTA can also effectively prevent iron from participating in the formation of the iron–doxorubicin complex, which is known to produce harmful reactive oxygen species that can cause damage to healthy tissues [33]. In various studies where EDTA counteracts the effects of anticancer drugs, it may serve as a valuable adjuvant therapy in preventing unwanted and potentially devastating effects in cases of accidental extravasation during the administration of anticancer drugs [33].

Regarding the issue of toxicity, the melanoma cell line C-32 and the epithelial adenocarcinoma cell line HeLa have been found to exhibit a moderate level of sensitivity to the chemical compound EDTA [33].

These carefully selected ingredients’ unique and beneficial properties ultimately prompted us to choose them as key components for developing the quercetin-based cream.

Quercetin is an extensively studied compound with well-documented anticancer properties, which are ascribed to its ability to modulate multiple cellular signaling pathways and inhibit enzymes involved in carcinogenesis. Furthermore, quercetin elicits its anticancer effects by interacting with cellular receptors and proteins [8,15,16,17,18,19]. The most distinctive biochemical characteristic of quercetin is its potent antioxidant capacity. The antioxidant activity and free radical scavenging properties of quercetin are directly related to its molecular structure [8,15,16,17,18,19,20].

Structural variables, encompassing molecular configuration, the degree of substitution, and the specific number of hydroxyl groups present, significantly influence the complex mechanisms involved in antioxidant activity. These multifaceted mechanisms include the ability to neutralize highly reactive radical species effectively and to selectively chelate metals that may otherwise participate in oxidative reactions [8].

Quercetin also effectively inhibits the lipid peroxidation process, a widespread consequence of oxidative stress, and consequently protects against damage to the lipid membrane. Due to its significantly lower redox potential, quercetin can successfully reduce the levels of highly oxidizing free radicals, such as superoxide and peroxide radicals. Due to its remarkable ability to chelate metal ions, quercetin can inhibit the generation of free radicals [8,20,21]. Understanding the bioavailability of quercetin, as well as the rate of metabolism and intestinal absorption, is significant in accurately describing the effectiveness of quercetin in terms of its anticancer effect [8]. Different forms of quercetin have different levels of bioavailability, and for some forms, like rutin, absorption can also depend on gender and the use of oral contraceptives [34]. In one study, when quercetin was administered intravenously to rodents, it immediately disappeared from the plasma, indicating rapid elimination. This experiment showed that quercetin was rapidly metabolized and eliminated from the body through urine, with no evidence of quercetin storage in tissues and bodily fluids. Previously, it was commonly hypothesized that quercetin was excreted in the feces without being absorbed by the intestine. Still, it is evident that an excessive amount of quercetin found in food is likely to be absorbed by the intestine and subsequently converted into respective metabolites. The lymphatic system is also significantly involved in transporting quercetin metabolites. In another study, repeated onion intake led to the accumulation of quercetin metabolites in various tissues and blood, reaching a total plasma concentration of 0.6 µM after a period of 1 week. Therefore, it is crucial to maintain the plasma concentration of quercetin metabolite at an acceptable and significant level [8,19,20].

Recent investigations have demonstrated that quercetin metabolites are rapidly distributed throughout various organs at low concentrations following prolonged dietary quercetin intake. Furthermore, it has been observed that regular consumption of quercetin-rich diets results in the accumulation of metabolites in tissues throughout the body. Although converting quercetin into its metabolic derivatives generally diminishes its free radical scavenging activity, specific quercetin metabolites can eliminate reactive species from the body. Notably, quercetin-3-glucuronide undergoes metabolism during inflammation, leading to quercetin aglycone accumulation [8,20,21].

Recent studies have revealed that glucuronide, a more active aglycone metabolite of quercetin, is incorporated into macrophages. This highlights the site-specific actions of quercetin metabolites, which are primarily recommended for inflammatory conditions. Modified forms of quercetin are present in human blood and stored in inactive forms. They are converted into active residues and ultimately transformed into active constituents that exert their effects at specific target sites. A diet rich in fruits and vegetables provides an abundance of phytochemicals with significant cancer-preventing potential due to their high antioxidant content [8,20,21].

A quercetin-based healing cream poses significant challenges because of the limited penetration of the compound through the skin, especially the stratum corneum (SC). The lipophilicity of quercetin means it will have a greater affinity for this stratum than for direct skin penetration [35]. However, melanoma lesions have a disrupted SC structure and reduced protection capabilities against external agents, possibly leading to higher local bioavailability in melanoma-affected skin, which works synergetically with glycerin and petroleum jelly to enhance the penetration capabilities of quercetin [35]. There have been studies that propose specific delivery systems for quercetin that would increase the relatively low bioavailability of quercetin (<10%) [36], such as solid dispersion-loaded dissolving microneedles [19]; incorporation into electrospun PAA NF via electrospinning [37]; encapsulation in cyclodextrins, conventional liposomes, and drug-in-cyclodextrin-in-liposomes [38]; and emulsions [39]. The most important aspect of using quercetin in a topical cream is the enhancement of penetration. This can be achieved using different emulsions formed with various compounds, such as Transcutol^®^ P, which has shown promise as a permeation enhancer for quercetin [40]. Another significant delivery mechanism is using solid lipid nanoparticles (SLNs). SLNs have been utilized to improve quercetin’s transdermal delivery. One study prepared quercetin-loaded SLNs using palmitic acid and varying surfactant ratios. The optimized formulation exhibited higher skin permeability than quercetin dissolved in propylene glycol, suggesting SLNs as a viable transdermal delivery system for hydrophobic antioxidants like quercetin [41]. Nanostructured lipid carriers (NLCs) have also been developed to enhance quercetin’s skin penetration. A study formulated quercetin-loaded NLCs with an average size of 130 nm. These nanoparticles demonstrated high entrapment efficiency (97.42%) and enhanced skin penetration, highlighting their potential as effective topical delivery systems for quercetin [42]. All these methods offer potential ways to increase the bioavailability of quercetin by transdermal delivery, addressing solubility and permeability challenges.

Another concern when using quercetin is the potential liver toxicity at a high dosage [43,44]. This is not necessarily a problem for topical application because of the limited bioavailability of quercetin, which is not significantly increased even if delivery systems are used. However, quercetin’s photodegradation is well-documented. When in the presence of ethanol, quercetin undergoes the addition of an ethanol molecule to the 2,3 double bond, accompanied by oxidation [45]. Direct evidence of its phototoxicity is limited because of the multiple possible interactions between excipients and quercetin. The formation of reactive oxygen species (ROS) during photodegradation could also theoretically lead to oxidative stress in skin cells, suggesting another potential phototoxic effect. Further research is necessary to elucidate the clinical significance of this potential phototoxicity.

Other natural compounds have also been studied for the treatment of melanoma. Studies have shown that polyphenols like quercetin possess antioxidant, anti-inflammatory, and anticarcinogenic properties in several in vitro and in vivo systems. One of these is epigallocatechin gallate (EGCG), which is found in green tea. EGCG inhibits melanoma cell growth by targeting multiple signaling pathways and inducing apoptosis [46,47]. Other natural compounds that have been studied include terpenoids [48] and curcumin, which can also act synergistically with quercetin [49]. Curcumin inhibits melanoma cell proliferation and metastasis. Research indicates that curcumin suppresses melanoma growth and metastasis through the miR-222-3p/SOX10/Notch signaling axis, highlighting its potential as a therapeutic agent against melanoma progression [50]. Similarly to quercetin, curcumin presents low bioavailability and requires diverse delivery mechanisms; however, it has also been more extensively studied.

## 5. Conclusions

Malignant melanoma, a highly aggressive form of cutaneous cancer, remains a significant clinical challenge due to its rapid progression and potential for metastasis. Early detection and prompt intervention are crucial for improving patient prognosis, as advanced-stage melanoma often limits therapeutic options. Treatment typically involves surgery followed by immunotherapy, targeted therapy, chemotherapy, and radiation, with adjunct therapies providing additional support.

This review has explored the potential of quercetin, a naturally occurring flavonoid, as an adjunct therapeutic agent in managing melanoma. Quercetin’s promising antiproliferative, antioxidant, and anticancer properties and ability to neutralize reactive oxygen species and modulate key carcinogenic pathways position it as a potential complementary agent in melanoma therapy. Based on its observed physicochemical properties and biological activity, quercetin could be developed into a topical formulation for cutaneous application, offering a novel approach to enhancing the effectiveness of conventional melanoma treatments.

A topical cream comprising quercetin; glycerin, a humectant that retains moisture and soothes the skin; petroleum jelly, a petroleum-derived ingredient that provides an occlusive barrier to prevent moisture loss; and EDTA, a chelating agent that enhances stability, exhibits considerable promise for promoting skin healing and protection by leveraging the synergistic effects of its components and can be considered for use as adjuvant therapy due to the ease of obtaining its ingredients and their low costs. However, the limitation presented by its poor bioavailability and toxicity at high doses or when exposed to light must be resolved with further research to determine the adverse effects of quercetin when used in a topical cream.

In summary, quercetin holds significant promise as an adjunct therapy for melanoma. Its multifaceted biological activities potentially target specific pathways involved in melanoma progression. Further clinical studies are needed to validate its efficacy as a topical cream and optimize its application with existing melanoma treatments.

## Figures and Tables

**Table 1:** Number of articles found by year for the second keyword combination.

SCOPUS		PUBMED
Year	Documents	Documents
2025	6	1
2024	20	14
2023	5	7
2022	22	6
2021	11	9
2020	8	8
2019	8	5
2018	5	5
2017	5	1
2016	4	3
2015	7	6

**Table 2:** Studies on the effect of quercetin on various cancer cell lines from the last 5 years.

Cancer Type	Study		Mechanism	Effect and Potential Application
	Authors and Title	Year		
Melanoma cells, SK-MEL-28 and G-361 lines, alongside non-tumorigenic HaCaT epidermal cells	Sang Young Seo et al. Quercetin Induces Mitochondrial Apoptosis and Downregulates Ganglioside GD3 Expression in Melanoma Cells [20]	2024	Inhibition of the FAK/paxillin/Akt signaling pathway.	Antiproliferative, antimigratory, and cell cycle arrest effects. Reduces the expression levels of ganglioside GD3.
Melanoma cells, human skin fibroblast cells (HFF-1 cells; SCRC-1041), and human malignant melanoma cells (A375 cells; CRL-1619)	Monsicha Khuanekkaphan et al. Development of Quercetin Solid Dispersion-Loaded Dissolving Microneedles and In Vitro Investigation of Their Anti-Melanoma Activities [19]	2024	It suppressed Bcl-2 gene expression.	Induction of cell apoptosis. Because of its increased solubility, the optimized Q-SD-DMN can be used in further in vivo studies as a synergistic method of melanoma treatment.
Keratinocytes (HaCaT) and melanoma (B16F10) cells	Anh Thu Ha et al. Anti-Inflammatory, Antioxidant, Moisturizing, and Antimelanogenesis Effects of Quercetin 3-O-β-D-Glucuronide in Human Keratinocytes and Melanoma Cells via Activation of NF-κB and AP-1 Pathways [21]	2021	Activation of NF-κB and AP-1 pathways.	Anti-inflammatory, antioxidant, moisturizing, and antimelanogenesis properties in human keratinocytes and melanoma cells. Bearing to glucuronic acid, quercetin can be used to protect skin cells.
Malignant melanoma cells, B16 and A375	Danhong Peng et al. Melanoma suppression by quercetin is correlated with RIG-I and type I interferon signaling [16]	2020	Quercetin upregulated IFN-α and IFN-β expression by activating RIG-I promoter in B16 cells. RIG-I likely amplifies antitumor effects by activating signal transduction and activator of transcription 1 (STAT1) in the IFN-JAK-STAT pathway in an autocrine and paracrine manner.	Quercetin inhibited mouse melanoma growth in vivo, suppressed proliferation, and promoted apoptosis.
MDA-MB-231 breast cancer cell line	Mohammadreza Roshanazadeh et al. Quercetin Enhances the Suppressive Effects of Doxorubicin on the Migration of MDA-MB-231 Breast Cancer Cell Line [28]	2021	Quercetin can affect the migration of MDA-MB-231 cells by reducing metastasis-related gene expression and significantly enhancing the inhibitory effects of doxorubicin on this expression.	Quercetin inhibits the viability and migration of MDA-MB-231 cancer cells and synergistically enhances the effects of dox on these cells’ survival and migration.
MCF-7 cells	Fatemeh Rezaie et al. Quercetin Arrests in G2 phase, Upregulates INXS LncRNA and Downregulates UCA1 LncRNA in MCF-7 Cells [29]	2022	Quercetin might increase cell death by upregulating INXS and downregulating UCA1 lncRNAs in MCF-7 cells.	Quercetin-induced cell cycle arrest at the G2 phase in MCF-7 cells.
Glioblastoma cells, T98G	Wanyu Wang et al. Quercetin induces MGMT+ glioblastoma cell apoptosis via dual inhibition of Wnt3a/β-Catenin and Akt/NF-κB signaling pathways. [30]	2023	Quercetin-induced apoptosis through decreasing MGMT expression. MGMT downregulation was achieved through dual inhibition of Wnt3a/β-Catenin and Akt/NF-κB signaling pathways.	Quercetin-induced S-phase arrest, DNA damage, and cell apoptosis.

## Data Availability

The original contributions presented in this study are included in the article. Further inquiries can be directed to the corresponding author(s).
